# Geographic determinants of reported human *Campylobacter *infections in Scotland

**DOI:** 10.1186/1471-2458-10-423

**Published:** 2010-07-15

**Authors:** Paul R Bessell, Louise Matthews, Alison Smith-Palmer, Ovidiu Rotariu, Norval JC Strachan, Ken J Forbes, John M Cowden, Stuart WJ Reid, Giles T Innocent

**Affiliations:** 1 Boyd Orr Centre for Population and Ecosystem Health, Institute of Comparative Medicine, Faculty of Veterinary Medicine, University of Glasgow, Bearsden Road, Glasgow, G61 1QH, UK; 2 Gastrointestinal Disease and Zoonoses, Health Protection Scotland, Clifton House, Clifton Place, Glasgow, G3 7LN, UK; 3 School of Biological Sciences, Cruickshank Building, St. Machar Drive, University of Aberdeen, Aberdeen, AB24 3UU, UK; 4 Section of Immunology and Infection, Medical School, University of Aberdeen, Foresterhill, Aberdeen, AB25 2ZD, UK

## Abstract

**Background:**

Campylobacteriosis is the leading cause of bacterial gastroenteritis in most developed countries. People are exposed to infection from contaminated food and environmental sources. However, the translation of these exposures into infection in the human population remains incompletely understood. This relationship is further complicated by differences in the presentation of cases, their investigation, identification, and reporting; thus, the actual differences in risk must be considered alongside the artefactual differences.

**Methods:**

Data on 33,967 confirmed *Campylobacter *infections in mainland Scotland between 2000 and 2006 (inclusive) that were spatially referenced to the postcode sector level were analysed. Risk factors including the Carstairs index of social deprivation, the easting and northing of the centroid of the postcode sector, measures of livestock density by species and population density were tested in univariate screening using a non-spatial generalised linear model. The NHS Health Board of the case was included as a random effect in this final model. Subsequently, a spatial generalised linear mixed model (GLMM) was constructed and age-stratified sensitivity analysis was conducted on this model.

**Results:**

The spatial GLMM included the protective effects of the Carstairs index (relative risk (RR) = 0.965, 95% Confidence intervals (CIs) = 0.959, 0.971) and population density (RR = 0.945, 95% CIs = 0.916, 0.974. Following stratification by age group, population density had a significant protective effect (RR = 0.745, 95% CIs = 0.700, 0.792) for those under 15 but not for those aged 15 and older (RR = 0.982, 95% CIs = 0.951, 1.014). Once these predictors have been taken into account three NHS Health Boards remain at significantly greater risk (Grampian, Highland and Tayside) and two at significantly lower risk (Argyll and Ayrshire and Arran).

**Conclusions:**

The less deprived and children living in rural areas are at the greatest risk of being reported as a case of *Campylobacter *infection. However, this analysis cannot differentiate between actual risk and heterogeneities in individual reporting behaviour; nevertheless this paper has demonstrated that it is possible to explain the pattern of reported *Campylobacter *infections using both social and environmental predictors.

## Background

Infection with bacteria of *Campylobacter *spp is the leading cause of human bacterial gastroenteritis in most developed countries [1]. In Scotland in 2006 there were 95.3 reported cases per 100,000 [2], although this figure is likely to represent only one in eight cases, as has been demonstrated in England [3]. Further studies in England and Wales show that approximately 10% of reported cases were admitted to hospital for treatment [4].

Infection with *Campylobacter *is thought to occur principally via the consumption of contaminated, under-cooked meat (mainly chicken) and cross-contaminated foods [5,6]. However other modes of transmission include direct and indirect contact with animal faeces (especially ruminant faeces) [7] and consumption of contaminated water [8-10]. Human exposure to these sources is spatially heterogeneous and therefore the spatial pattern of infection is heterogeneous.

Previous studies have identified risk factors that include eating chicken, eating in restaurants and eating from fast food outlets [6,11]. Additionally, those who live in rural areas and have regular contact with livestock are at greater risk of infection [12-15], as are individuals with private water supplies [10,11]. Further variations in *Campylobacter *incidences caused by either physiology or differences in exposure relate to the age and gender of the individual. For example, male children are at around 1.5 times greater risk of infection than their female counterparts [16,17].

In addition to heterogeneity in infection there will be heterogeneity in reporting. Infections may be under ascertained by a factor of 8 [3], but this may not necessarily be distributed evenly throughout the population. Reporting may be influenced by the age and gender of the patient [14,16], use of primary health care facilities [18,19] and the socio-economic status of the patient [20].

This study developed a risk factor model to explain the geographical distribution of *Campylobacter *infections incorporating both sources of heterogeneity - risk of infection and risk of reporting. At the level of the community it distinguished between factors that determine risk of *Campylobacter *infections and factors that determine artefactual risk due to reporting differences between NHS Health Boards. Thus the study provides an overall model of the geographic pattern of reported *Campylobacter *cases within Scotland. The study has the following aims:

1. To quantify the importance of deprivation in determining *Campylobacter *infections given that deprivation may influence food consumption, environmental contact and propensity to seek medical attention or submit a stool sample.

2. To identify rural-urban differences in *Campylobacter *infections and whether such differences may be explained by proximity to livestock.

3. To identify differences in *Campylobacter *infections between NHS Health Board areas.

4. To establish whether these differences are age dependent.

## Methods

### Data

Data on cases of *Campylobacter *infection were collected by staff at Health Protection Scotland (HPS) from the public health teams at the 12 mainland NHS Health Boards that existed in Scotland prior to 2006. Ethical approval for the collection and use of the data was obtained from the Multi-Centre Research Ethics Committee (MREC) in Scotland; approval for the research was also obtained from the Research and Development Committee in each of the NHS Health Boards. Data for the island NHS Health Boards of the Western Isles, Orkney and Shetland were not collected due to their small populations and small numbers of cases. Data were collected for the years 2000 to 2006 (inclusive), with the exception of the Ayrshire & Arran NHS Health Boards for which only the years 2003 to 2006 (inclusive) were available. Data were anonymised and included the age, gender and postcode sector of residence of the case, and the date of reporting. Subsequently all analysis was at the level of the postcode sector (median population = 5,977, 25^th, ^and 75^th ^percentile = 3,788 and 7,847; median area = 12.4 km^2^, 25^th,^and 75^th ^percentile = 2.23 and 75.2 km^2^).

Data on the human population were collected from the 2001 Scottish census [21] along with data on the Carstairs index of deprivation; cattle, sheep and poultry numbers were obtained from the Scottish agricultural census [21] (2004 estimates). Data on recent travel was available for the Lothian and Grampian NHS Health Board areas from the Health Protection Scotland (HPS) enteric disease reporting forms.

### Risk factors

The following risk factors were included for initial screening:

• The Carstairs deprivation score [22].

• Easting and northing of the postcode sector centroid.

• Population density (people/km^2^) of the postcode sector (log_10 _transformed to linearise its relationship with the response mean on the log-scale).

• Density of cattle, sheep and poultry (head/km^2^) in the postcode sector.

### The model

The risk factors listed above were screened in univariate generalised linear models (GLM) with a Poisson distributed outcome and those with p < 0.25 selected for insertion into the multivariable model. The multivariable model was a spatial generalised linear mixed model (GLMM) in which the spatial structure was modelled as a Gaussian Markov Random Field (GMRF) and the model was fitted using the Integrated Nested Laplace Approximation (INLA) method [23]. In the case of this model the GMRF is the neighbourhood dependency, incorporated by including the network of neighbouring postcode sectors as a random effect. Thus the GMRF allows for the fitting of a spatial conditional autoregressive random effect that accounts for the spatial dependency when fitting the model, as described by Besag et al [24]. The outcome (number of cases) was fitted with a Poisson distribution that was offset by the log of the population of the postcode sector (*O_j_*). Thus, the model takes the form:

$$\begin{array}{l} Y_{ij}\sim Poisson(\lambda_{ij})\\ \log(\lambda_{ij})=\beta^{T}X_{ij}+H_{i}+U_{ij}+V_{ij}+\log(O_{ij}) \end{array}$$

where *H_i _*represents the effect of health board *i*; *V_ij _*the spatially structured variation associated with being in postcode sector *j *in health board *i *and *U_ij _*the corresponding unstructured variation. *X_ij _*represents the vector of risk factors in each postcode sector in each health board. The mean, 2.5% and 97.5% quantiles of the estimated coefficients were used to calculate mean relative risks (RRs) and the 95% confidence intervals of the RRs. INLA was implemented in the INLA package [25] for the R statistical environment [26]. The model fit was checked by inspecting the mean, 2.5% and 97.5% quantiles of the posterior distributions of the standard deviations (sd) of the random effects. Large, or asymmetrical 95% intervals would indicate poor model fit.

Data were gathered from the 12 mainland NHS Health Board areas. However, for these analyses this was converted to a factor with 13 levels by breaking the Argyll & Clyde NHS Health Board into separate Argyll and Clyde areas. The Clyde area was defined by the area straddling the mouth of the Clyde and the remainder of the Argyll & Clyde NHS Health Board as Argyll. This was due to the ten-fold difference in case rates between the two (Table 1; with highly significant (p < 0.001) differences across the boundary - unpublished data). Similar divisions were not found within other NHS Health Boards (unpublished data).

**Table 1:** Summary statistics for NHS Boards.

NHS Health Board	Population	Number of postcode sectors	Population density	Total number of cases	Cases/100000/yr
Argyll & Clyde (AC)	414,991	76	55.0	2,308	79.5
*Argyll (AR)*	*84,067*	*26*	*12.1*	*56*	*9.5*
*Clyde (CL)*	*330,924*	*50*	*531.5*	*2,252*	*97.2*
Ayrshire & Arran (AA)	367,073	69	108.9	750	51.1
Borders (BR)	103,943	26	23.4	777	106.8
Dumfries & Galloway (DG)	147,625	34	22.9	1,214	117.5
Fife (FF)	346,391	49	265.5	1,762	72.7
Forth Valley (FV)	281,747	44	100.4	2,040	103.4
Grampian (GR)	524,337	99	59.9	5,104	139.1
Greater Glasgow (GG)	867,394	133	1539.4	4,984	82.1
Highland (HG)	194,139	55	7.4	1,280	94.2
Lanarkshire (LN)	552,397	62	221.3	4,086	105.7
Lothian (LO)	775,874	116	469.2	7,250	133.5
Tayside (TY)	384,644	67	51.6	2,412	89.6

**Total**	**4,960,556**	**830**	**67.9**	**33,967**	**101.0**

To allow for the differences in the ages of cases [16] and to test for the age dependent differences in the effect of rurality noted in Denmark [14], separate models were constructed for those aged under 15 years and those 15 and over (the cut-off at 15 was selected due to five year age groupings in the Scottish census data). The relative risk estimates for the final model including population density (irrespective of whether it a was included in the final model) were compared for those aged under 15 years, 15 and over, and all data.

Further sensitivity analysis was conducted by building models for just the Lothian and Grampian NHS Health Board areas and running the model with and without the cases that had travelled overseas in the previous 14 days. The Lothian and Grampian NHS Health Boards were selected because it was only these Boards for which overseas travel data was available. Evaluation of the relative change in the model coefficients indicates whether the model results were a result of foreign travel. The RRs for the risk factors in these models were compared.

## Results

### NHS Board differences

**Figure 1:**
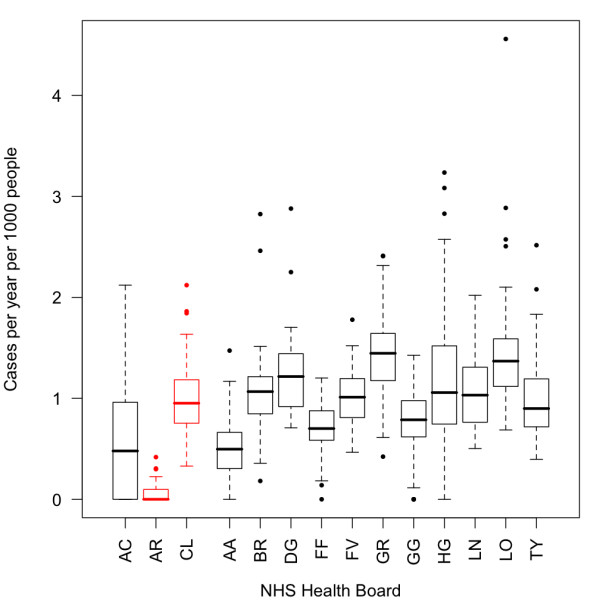
**Boxplot of case rates by NHS Health Board**. Boxplot of case rates per year for the 12 NHS Boards (in black) and the Argyll and Clyde NHS Board divided into in the separate units (in red). NHS Board abbreviations are expanded in Table 1.

A total of 33,967 cases were reported over the course of the study period. There was a ten-fold difference in the rates reported in the Argyll relative to the Clyde area of the Argyll & Clyde NHS Health Board (Table 1). Case rates in the Ayrshire & Arran NHS Health Board were around 50% lower than those seen in other NHS Health Board areas (Figure 1).

**Figure 2:**
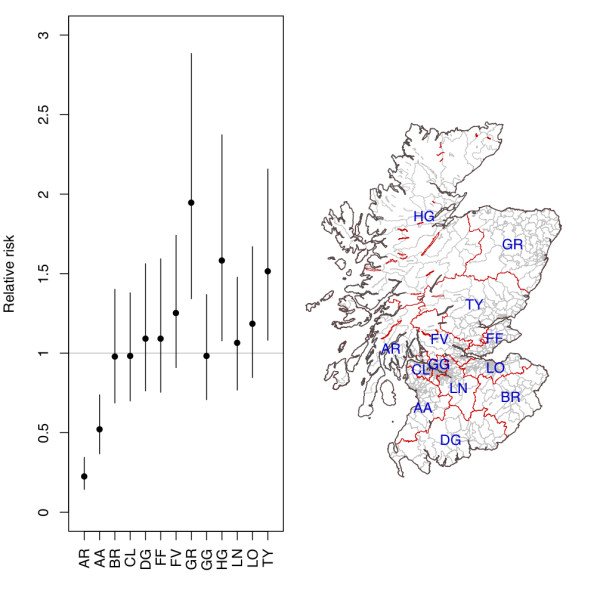
**Effect and distribution of NHS Health Board**. RRs and 95% CIs attached to each NHS Health Board from the model and the geographical distributions of the health boards (red borders) relative to postcode sectors (grey borders).

Once the other risk factors have been taken into account, the Argyll sector of the Argyll & Clyde NHS Health Board and the Ayrshire & Arran NHS Health Board remain significantly lower than expected (Figure 2). Despite having the second highest case rate (Table 1, Figure 1), the RR of infection in the Lothian Health Board is not significantly different from one (Figure 2). However, the Grampian, Highland and Tayside NHS Health Boards are significantly greater than one (Figure 2).

### Risk factor analysis

All risk factors with the exception of sheep and poultry densities were significant at p < 0.25 in univariate screening (Table 2). In the reduced multivariable model, the Carstairs deprivation index and the population density were significant and retained (Table 3). Greater deprivation and greater population densities were associated with lower case incidences. In addition, the standard deviations of posterior distributions of the random effects were normally distributed (Table 3). Sensitivity analysis for foreign travel in the Lothian and Grampian NHS Health Board areas indicate that there is no sensitivity to foreign travel as there was no significant difference in the model coefficients with and without the cases who have undertaken recent foreign travel.

**Table 2:** Univariate poisson GLM analysis of risk factors.

Predictor	Unit	Estimate	Std. error	t-value	p-value
Easting	km	3.533 × 10-3	2.457 × 10-4	14.38	< 0.001
Northing	km	9.412 × 10-4	1.835 × 10-4	5.13	< 0.001
Carstairs score		-0.050	0.004	-14.23	< 0.001
Human density	log_10_(people/km^2^)	-0.031	0.015	-2.059	0.040
Cattle density	cattle/km2	-6.401 × 10-4	3.854 × 10-4	-1.661	0.097
Sheep density	sheep/km2	-4.655 × 10-6	1.631 × 10-4	-0.029	0.977
Poultry density	poultry/km2	-2.460 × 10-6	3.654 × 10-6	-0.673	0.501

**Table 3:** Posterior distributions of the fitted terms in the reduced spatial GLMM for *Campylobacter *risk.

Predictor	Measure	Unit	Mean (95% CIs)
Intercept	Estimate		-6.893 (-7.209, -6.582)
**Risk factors**			
Carstairs	Relative risk		0.965 (0.959, 0.971)
Population density	Relative risk	log_10_(people/Km^2^)	0.945 (0.916, 0.974)
**Random effects**			
V	Posterior sd		0.249 (0.213, 0.296)
U	Posterior sd		0.082 (0.060, 0.116)
H	Posterior sd		0.502 (0.356, 0.788)

### Age-stratified analysis

**Figure 3:**
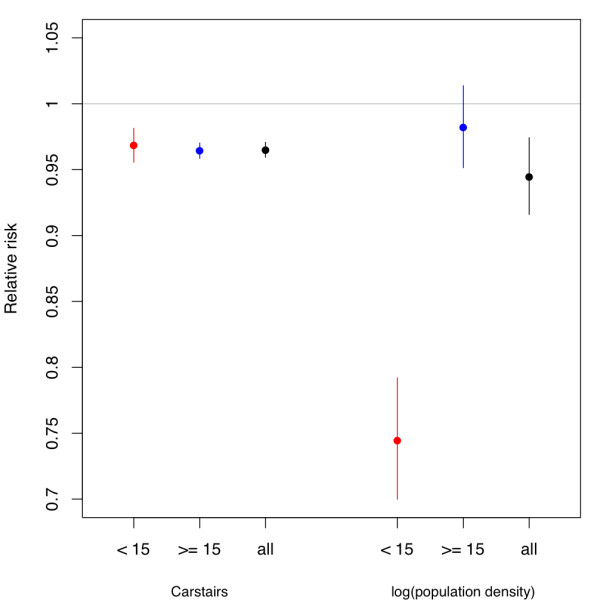
**Plot of the age dependence in population density**. RRs and 95% CIs for the fixed effects in the model presented in Table 3 separately fitted using data on cases under 15 years old, 15 and over and all data.

Comparison of the coefficients for the Carstairs deprivation score and population density amongst individuals aged under 15 years compared to 15 and over shows that there is little change in the RR for the Carstairs deprivation index (Figure 3). However, there is a significant protective effect of population density in those aged under 15 (RR = 0.745, 95% CIs = 0.700, 0.792), compared to the non-significant effect (RR= 0.982, 95% CIs = 0.951, 1.014) in individuals aged 15 and over (Figure 3).

## Discussion

Reported *Campylobacter *infections are more common among the least deprived and amongst children living in rural areas. This could be a result of real differences in rates of infection or due to differences in ascertainment. These results are in line with findings from other countries for both *Campylobacter *and other gastrointestinal diseases [18,20]. A number of potential explanations have been offered for the relationship with deprivation:

1. Acquired immunity through exposure to household sources of infection at a young age amongst the more deprived. The level of exposure among younger age groups to bacterial sources of infection within the household may increase with deprivation. However, Figure 3 demonstrates that there is no significant difference in the Carstairs deprivation score in the age-stratified analysis. If acquired immunity were the explanation then the younger groups would be more commonly infected in more deprived areas whilst older age groups would be more commonly infected in less deprived areas, however, Figure 3 does not support this. These findings are supported by other studies that suggest that there is no difference between age and deprivation [18,20].

2. Deprivation may be associated with differences in dietary habits [18]; differences in the quality of the available fresh food have been observed elsewhere [27]. If there is greater consumption of processed rather than fresh meat among the more deprived there will be less *Campylobacter *because the process of freezing reduces the number of *Campylobacter *organisms [28]. Furthermore, the less deprived may also eat at restaurants more frequently, which has been demonstrated as a risk factor in other studies [11].

3. Differences in environmental exposure associated with different leisure activities, differences in access to rural areas or people working in rural areas.

4. Differences in reporting. Lower reporting rates for gastrointestinal disease among the more deprived have been noted in the UK [19,29,30], Denmark [18] and New Zealand [29,30].

5. Differences in foreign travel. The sensitivity analysis, however, in the two NHS Health Board areas for which travel data were available indicated that this is not the case.

Further research is necessary to fully understand the processes operating, for example comparing hospitalisation rates; however, it is likely that some combination of these factors is responsible for the relationship with deprivation.

The significance of the protective effect of population density among children confirms findings from Denmark [14] where significantly higher case rates were found among children in rural areas. This may be the result of differences in the tolerance level that determines whether a patient reports to a doctor, which is likely to be age dependant. Alternatively, or in combination, that children in rural areas are playing outdoors and becoming exposed to environmental reservoirs of infection, and may additionally be compounded if there is poorer hygiene among younger groups.

Whilst one of the greatest sources of *Campylobacter *in rural areas is likely to be livestock [7,31,32], our analysis did not show density of livestock to be associated with *Campylobacter *infections. Furthermore, contamination of private water supplies [10,11], which is associated with low population density, may be an additional source of infection. Therefore, these findings suggest that environmental exposures, whilst these may ultimately be the result of contamination from livestock sources, are best characterised by low population densities.

The model incorporated the spatial structure of the data because it can not be assumed that the data are spatially independent as neighbouring postcode sectors may have similar properties. Whilst most exposure to infection is likely to occur in the postcode sector of residence, the incorporation of the spatial structure allows for environmental exposures to infection arising from travel outside of the postcode sector of residence.

Once these predictors were taken into account, differences were noted between NHS Health Board areas, in particular, the Argyll area of the Argyll & Clyde NHS Health Board area and the Ayrshire and Arran Health Board that had RRs significantly lower than 1. This suggests that after the factors in the model have been taken into account there remains some mechanism affecting incidence or reporting of *Campylobacter *infection. In addition, the Ayrshire & Arran NHS Health Board area also reported less GI disease per head of population than any other NHS Health Board area in Scotland for both *Salmonella *and *Cryptosporidium *infections (unpublished data). Furthermore, several NHS Health Boards have significantly higher case incidences, in particular the Grampian, Highland and Tayside NHS Health Boards. This may be the result of some factors not included in the model or reporting differences. However, the Lothian NHS board that has the second highest case incidence is not significantly different from expected in the final model, suggesting that in this Health Board the other factors in the model explain the patterns of reporting in this district.

## Conclusions

This study has demonstrated that there are real differences in the geographic distribution of *Campylobacter *infections within Scotland, which are either caused by differences in exposure to infection, or differences in individuals reporting infection. Variation due to reporting at the level of the Health Board has been accounted for in the model. Those at greatest risk are the less deprived and children in rural environments. The results suggest that the relationship with deprivation is unlikely to result from differences in acquired immunity. Furthermore, those less deprived may be more exposed to infection or may be more willing to seek medical attention. However, large differences remain in reported disease incidences between the deprived and the less deprived as well as differences in ascertainment between the boards administering the health care.

## Competing interests

The authors declare that they have no competing interests.

## Authors' contributions

PRB carried out data analysis and drafted the manuscript. LM participated in the design and guidance of the study and drafting of the manuscript. ASP and JMC gathered, participated in the cleaning and processing of the *Campylobacter *case data and assisted with specific public health aspects of the study. OR, NJCS and KJF participated in the cleaning and processing of the data on risk factors and assisted with biological aspects of the study and carried out detailed analyses of heterogeneities within and between Health Boards. SWJR participated in the design and coordination of the study. GTI carried out initial data processing, assisted with statistical analysis and participated in the drafting of the manuscript. All authors read and approved the final manuscript.

## Pre-publication history

The pre-publication history for this paper can be accessed here:

http://www.biomedcentral.com/1471-2458/10/423/prepub
